# Toward Bioactive Hydrogels: A Tunable Approach via Nucleic Acid-Collagen Complexation

**DOI:** 10.1007/s40883-024-00345-1

**Published:** 2024-05-28

**Authors:** Nikolaos Pipis, Senthilkumar Duraivel, Vignesh Subramaniam, Kevin A. Stewart, Thomas E. Angelini, Josephine B. Allen

**Affiliations:** 1 J. Crayton Pruitt Family Department of Biomedical Engineering, University of Florida, Gainesville, FL 32611, USA; 2 Department of Materials Science & Engineering, University of Florida, Gainesville, FL 32611, USA; 3 Department of Mechanical and Aerospace Engineering, University of Florida, Gainesville, FL 32611, USA; 4 George & Josephine Butler Polymer Research Laboratory, Department of Chemistry, Center for Macromolecular Science & Engineering, University of Florida, Gainesville, FL 32611, USA

**Keywords:** DNA-collagen complexes, Tunable biomaterials, Microfibers, Aptamers, Collagen, Rheology

## Abstract

**Purpose:**

Nucleic acid-collagen complexes (NACCs) are unique biomaterials formed by binding short, monodisperse single-stranded DNA (ssDNA) with type I collagen. These complexes spontaneously generate microfibers and nanoparticles of varying sizes, offering a versatile platform with potential applications in tissue engineering and regenerative medicine. However, the detailed mechanisms behind the nucleic acid-driven assembly of collagen fibers still need to be established. We aim to understand the relationship between microscopic structure and bulk material properties and demonstrate that NACCs can be engineered as mechanically tunable systems.

**Methods:**

We present a study to test NACCs with varying molar ratios of collagen to random ssDNA oligonucleotides. Our methods encompass the assessment of molecular interactions through infrared spectroscopy and the characterization of gelation and rheological behavior. We also include phase contrast, confocal reflectance, and transmission electron microscopy to provide complementary information on the 3D structural organization of the hydrogels.

**Results:**

We report that adding DNA oligonucleotides within collagen robustly reinforces and rearranges the hydrogel network and accelerates gelation by triggering rapid fiber formation and spontaneous self-assembly. The elasticity of NACC hydrogels can be tailored according to the collagen-to-DNA molar ratio, ssDNA length, and collagen species.

**Conclusion:**

Our findings hold significant implications for the design of mechanically tunable DNA-based hydrogel systems. The ability to manipulate hydrogel stiffness by tailoring DNA content and collagen concentration offers new avenues for fine tuning material properties, enhancing the versatility of bioactive hydrogels in diverse biomedical applications.

**Lay Summary:**

This work is an example of forming fibers and gels with tunable elasticity that stems from the complexation of short-length nucleic acids (on the order of size of aptamers) and collagen, which can be potentially extended to a variety of functionalized hydrogel designs and tailored biomedical applications. Incorporating DNA induces mechanical changes in NACCs.

## Graphical Abstract

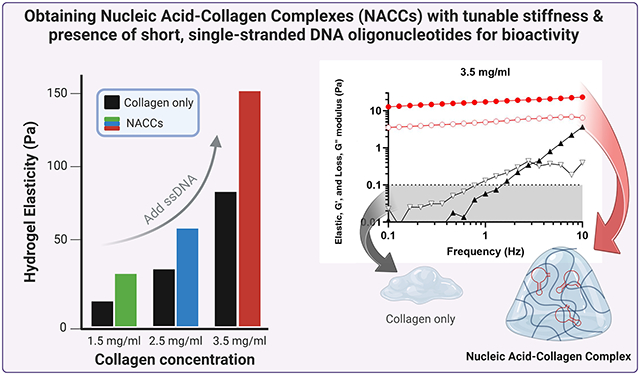

## Introduction

Over the years, significant advancements in hydrogel technology have propelled the biomaterials field forward, enabling the development of increasingly sophisticated and tailored hydrogel systems for a wide range of biomedical applications. Among the many material systems under investigation, those derived from natural biological sources have been widely studied due to their inherent biocompatibility and their ability to replicate the native extracellular matrix (ECM) environment. This is particularly true for collagen-based hydrogel materials. Collagen is an attractive material to explore in biomedical applications, being the most abundant protein contained within human connective tissues and natural ECM. Collagen-based hydrogels can be fabricated through self-assembled fibrillogenesis of collagen monomers, which in turn assemble further into a triple-helical protein network, further assembled into larger fibrils and fibers with lengths on the order of around 10 μm [1]. This larger, hierarchical fibrillary network has characteristic material properties that closely match tissue-specific native ECM. Collagen hydrogels have been studied and characterized extensively and have shown promise in diverse biomedical applications, such as tissue engineering [2], drug encapsulation and delivery [3], biosensing [4], and applications with tissue models, to name a few.

Despite the progress made in the field, there is still a need for strategies that offer precise control over hydrogel properties while maintaining biocompatibility, biomimicry, and reproducibility. While synthetic hydrogels are often used in three-dimensional cell culture, their complex chemistries, inconsistent gelation, and reliance on biologics for cellular support pose challenges [5–7]. In some cases, translating synthetic hydrogels in vivo requires specialized equipment and can result in cell necrosis, inflammation, and fibrosis. In this context, using short (< 100 nucleotides) single-stranded DNA oligonucleotides as a tunable component within a collagen-based hydrogel represents a highly promising and unexplored approach. As a highly hydrophilic polyelectrolyte, DNA can be programmed through base pair interactions, is biocompatible, and has target recognition properties [8]. DNA self-hybridization and DNA origami techniques have been extensively studied [9], but their application as tunable factors in hydrogels remains relatively uninvestigated. Approaches involving DNA oligonucleotides being used to crosslink other hydrogel systems, such as a polyacrylamide featuring reversible DNA crosslinks, have been reported [10]. This replaces traditional chemical crosslinkers and allows for both crosslink formation and dissociation without the need for external heat, achieved through the addition of complementary base sequences to the crosslinker sequence. Another approach highlights the integration of aptamers in natural protein-based polymers, showcasing the potential of combining structural and functional features to enhance hydrogel performance [11]. These advances underscore the growing recognition of DNA’s role in influencing hydrogel behavior and its potential for tailoring material properties and bioactive functionalities.

The complexation of DNA with collagen, termed nucleic acid-collagen complexes or NACCs, has been previously reported by our group to represent a versatile biomaterial platform with significant potential for tissue engineering and regenerative medicine applications. We reported that NACCs are self-assembled microfibrous structures formed through the complexation of single-stranded DNA (ssDNA) oligonucleotides or sequence-specific DNA aptamers with type I collagen. The formation of NACCs is independent of the specific ssDNA sequence, but it is influenced by the length of the ssDNA and the ratio of ssDNA to collagen in solution. Shorter ssDNA aptamers (< 100 nucleotides) have been shown to form microfibers [12]. These complexes exhibit rapid and spontaneous formation at room temperature, requiring no additional treatment, and when mineralized, closely resembling the architecture of native ECM, with pits, pores, and striations, making them promising candidates for tissue engineering applications [13, 14]. Incorporating specific bioactive DNA aptamers into NACCs presents an opportunity to confer site specificity and enhance the functionality of these complexes. Additionally, NACCs fabricated with a vascular endothelial receptor-2 agonist aptamer have also shown enhanced expression of key angiogenic factors, indicating their potential for promoting angiogenic endothelial cell phenotypes [15].

NACCs offer a promising and biofunctional natural scaffold material system. Further research into the interplay between DNA aptamers and collagen in NACCs will deepen our understanding and expand the potential of this biomaterial platform. This report represents a novel exploration of NACCs, aiming to harness the interaction of collagen with DNA to test characteristics such as gelation kinetics and elastic modulus and observe their microstructural composition. In this study, we explore the tunability of NACC hydrogels with varying collagen-to-DNA molar ratios, providing a greater understanding of the NACC system. We give valuable insights into the interplay between DNA and collagen in controlling gel properties. Through FTIR-ATR, rheology, and microscopy, we shed light on the variabilities between collagen only and NACCs, paving the way for developing advanced biomaterials with precise control over their properties and functionalities. We also demonstrate the fine tuning of NACCs by adjusting the relative length of the ssDNA sequence and testing with collagen derived from different species.

## Materials and Methods

### Sample Preparation

NACC fibers were fabricated by mixing different concentrations (1.5 mg/mL, 2.5 mg/mL, 3.5 mg/mL) of type 1 bovine collagen (Advanced Biomatrix PureCol EZ Gel, molecular weight = 300 kDa) diluted in sterile deionized water, with 10 μM of ssDNA oligomers of different lengths (Integrated DNA Technologies) diluted in sterile deionized water (Table 1). Complexation occurs at room temperature simply by pipetting the two solutions together at the desired ratios.

We investigated different hydrogel compositions and their related mechanical properties by varying the molar ratio of collagen to ssDNA (Table 2). For controls, the same process was repeated with deionized water without any resuspended ssDNA (i.e., samples contained collagen only at the desired concentrations). To confirm and verify our findings with collagen derived from other species, we conducted the same experiments using type 1 rat-tail collagen (Corning).

### FTIR-ATR

Infrared spectra were acquired on a PerkinElmer Spectrum One FTIR spectrometer equipped with a PIKE MIRacle single-reflection ATR accessory containing a diamond crystal sample plate. Spectra were processed using PerkinElmer Spectrum 10 software. Spectra were recorded with 64 scans within a spectral window of 4000–650 cm^−1^, with the bands of interest occurring between 2000–650 and 3000–3500 cm^−1^. A background of sterile deionized water was collected and subtracted prior to collagen and NACC sample analysis. A type 1 bovine collagen control sample was loaded onto the ATR diamond with a total volume of 40 μL (final concentration 2.5 mg/mL). The NACC sample was loaded onto the ATR diamond by adding 20 μL of type 1 bovine collagen followed by 20 μL of ssDNA. The complex was allowed to incubate for approximately 30 min prior to spectral analysis.

### Rheological Analysis

Using the MCR702 rheometer (Anton Paar), we set a gap size of 0.5 mm between sandblasted, 25 mm diameter parallel plates to ensure uniform and controlled sample thickness. The temperature was set to 25 °C. Collagen was held in an ice chamber until being transferred to the rheometer. Working quickly, collagen was pipetted onto the lower rheometer plate, followed by adding the resuspended ssDNA and pipetting further, taking care to avoid introducing air bubbles. For consistency, inhomogeneities in the gel matrix were carefully prevented by pipetting up and down several times while stirring with the pipet tip. Immediately after, the upper plate was lowered to create a uniform hydrogel layer between the parallel plates. We ensured proper hydration of the area around the sample to prevent excessive evaporation.

Gelation was assessed by measuring the increase in modulus over time while oscillating at a constant frequency of 1 Hz and a strain amplitude of 3% (within the linear viscoelastic region). The storage modulus (*G*′) and loss modulus (*G*″) were monitored over a 45-min time period to capture the evolution of the gel network. Measurements were performed at the specified low frequency and small strain amplitude to ensure accurate detection of gelation and prevent sample damage.

Following gelation, small amplitude frequency sweep measurements were performed on the now mature DNA collagen gels, applying an oscillatory shear at varying frequencies over a defined range of 0.1 to 10 Hz and a strain amplitude of 3%. *G*′ and *G*″ were measured at each frequency, providing information about the gel’s ability to store and dissipate energy.

### Statistical Analysis

Rheological data was analyzed via Prism 9 (GraphPad). Each sample included three replicates (*n* = 3). Statistical analysis was conducted for the frequency sweep values to assess the significant differences among the means of samples using a two-tailed *t*-test with a 95% confidence interval.

For calculating gelation time, *t*_*gel*_, we fit a logistic regression model to the elastic shear modulus, *G*′, as a function of time, *t*, based on the Hill equation [16], given by

$$G\prime \left(t\right)=G_{0}-\frac{G_{o}}{1+{\left(\left(t+t_{0}\right)/t_{\text{gel}}\right)}^{p}}$$

Here, *G*_*o*_ is the modulus of the fully crosslinked gel*, t*_*gel*_ is the time required to achieve half of the plateau modulus (gelation time), *t*_0_ reflects the delay time between sample mixing and the start of the measurement, and *p* is a dimensionless number.

### Imaging

The goal was to evaluate the differences between type 1 collagen with and without the addition of DNA oligonucleotides, as well as confirm the presence of fibers in NACCs.

Phase contrast images were taken using the Keyence BZ-X800 and a PlanFluor 20 × 0.45/8.80–7.50 mm lens.

To visualize the internal structure of the hydrogels through confocal reflectance microscopy (CRM), samples were prepared in glass bottom dishes and allowed to gel. CRM was performed on a Nikon Eclipse TI with a × 60 objective and immersion oil. Z-stacks were obtained with dimensions of 212.13 μm (width) × 212.13 μm (height) × 80 μm (depth). The images along the *z*-axis were then reconstructed to give a 3D visualization of the hydrogel’s network organization.

For transmission electron microscopy (TEM), the Talos L120C G2 was used. Samples were placed on glow discharged, carbon coated Formvar copper grids, 400 mesh, and stained with 2% uranyl acetate for subsequent negative stain imaging. The images were acquired on a CETA camera (Thermo Fisher Scientific).

### Image Processing

We used Fiji (ImageJ version 2.14.0) for CRM to visualize the hydrogels’ three-dimensional features from Nikon nd2 files. The images were first denoised using a Gaussian blur filter, and automated fiber segmentation was performed with the ridge detection plugin in Fiji. For measuring the fiber diameters, the optimal parameters for each sample were determined by varying the line width and length, followed by a visual review of the detected segments using the preview function in Fiji (Supplementary Fig. 1).

## Results

### Interaction Between Collagen Peptides and ssDNA

**Fig. 1:**
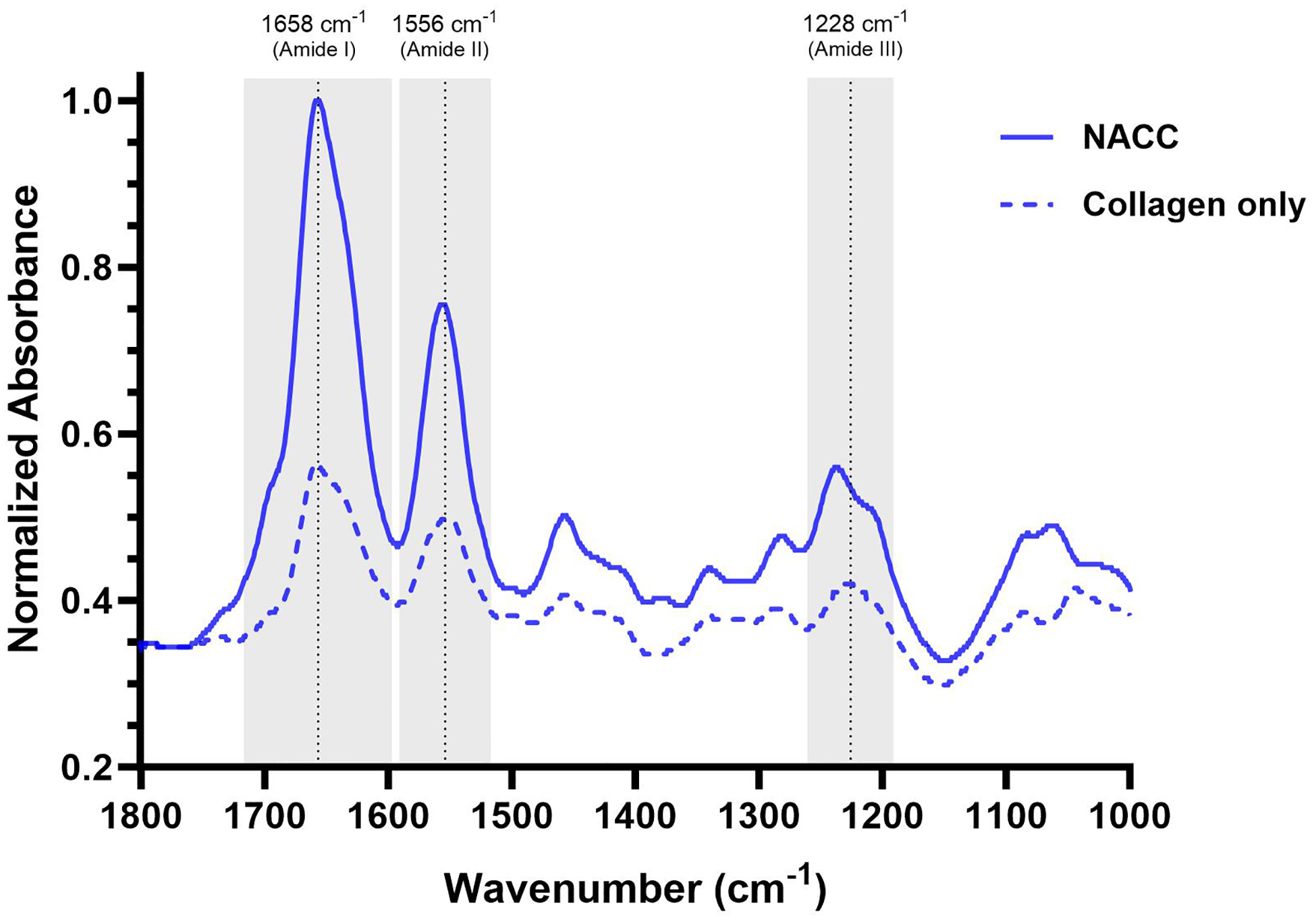
FTIR-ATR spectra of collagen only (dotted lines) and NACC (solid line). The spectra highlight bands of interest at 1658, 1556, and 1228 cm^−1^ (corresponding to amide stretching of collagen) as well as 1280 cm^−1^ (related to the sugar-phosphate backbone stretching of ssDNA)

FTIR-ATR spectroscopy was utilized to qualitatively assess the molecular interaction between collagen fibrils and the ssDNA (Fig. 1). We first collected the spectrum of type 1 bovine collagen to establish a characteristic baseline of the virgin polypeptide. The IR spectrum displayed three major bands at approximately 1658, 1556, and 1228 cm^−1^, characteristic of the amide I (C=O stretching), amide II (N–H bending), and amide III (C–N stretching) within the polyamide backbone, respectively [17, 18]. Next, equal volumes of type 1 bovine collagen and ssDNA were added sequentially onto the ATR diamond and allowed to incubate for approximately 30 min to ensure complete complexation and fibro-genesis. The IR spectrum was then collected, revealing not only the retainment of the amide bands at 1658, 1556, and 1228 cm^−1^ but also a notable amplification of these signals. We attribute this signal augmentation to the increased localized concentration of collagen fibrils following complexation with ssDNA, agreeing well with TEM images of the NACC (Fig. 6) and the distinctive increase in opacity in the aqueous dispersions following complexation. Furthermore, we noted the appearance of a distinguishable peak at approximately 1280 cm^−1^ that was not clearly evident in the collagen-only sample. This band is attributed to the sugar-phosphate backbone of the DNA, which is in good agreement with literature precedent [19]. However, the position of the amide A and B bands in the region 3200–3500 cm^−1^, attributed to N–H stretching of the secondary amide, also remained unchanged with no distinguishable shifts in peak position (Supplementary Fig. 2). As such, there is no clear evidence for a strong hydrogen-bonding interaction as the driving force for complexation, which would clearly manifest as not only large frequency shifts but also typically increases in band intensity. This lack of significant hydrogen-bond bridging between species may suggest that complexation is solely based on supramolecular interactions driven by hydrophobic interactions of the collagen and ssDNA [20]. Nevertheless, the presence of identical bands reveals no chemical changes occurring upon complexation nor alterations in collagen’s secondary structure.

### ssDNA Accelerates NACC Gelation Kinetics

**Fig. 2:**
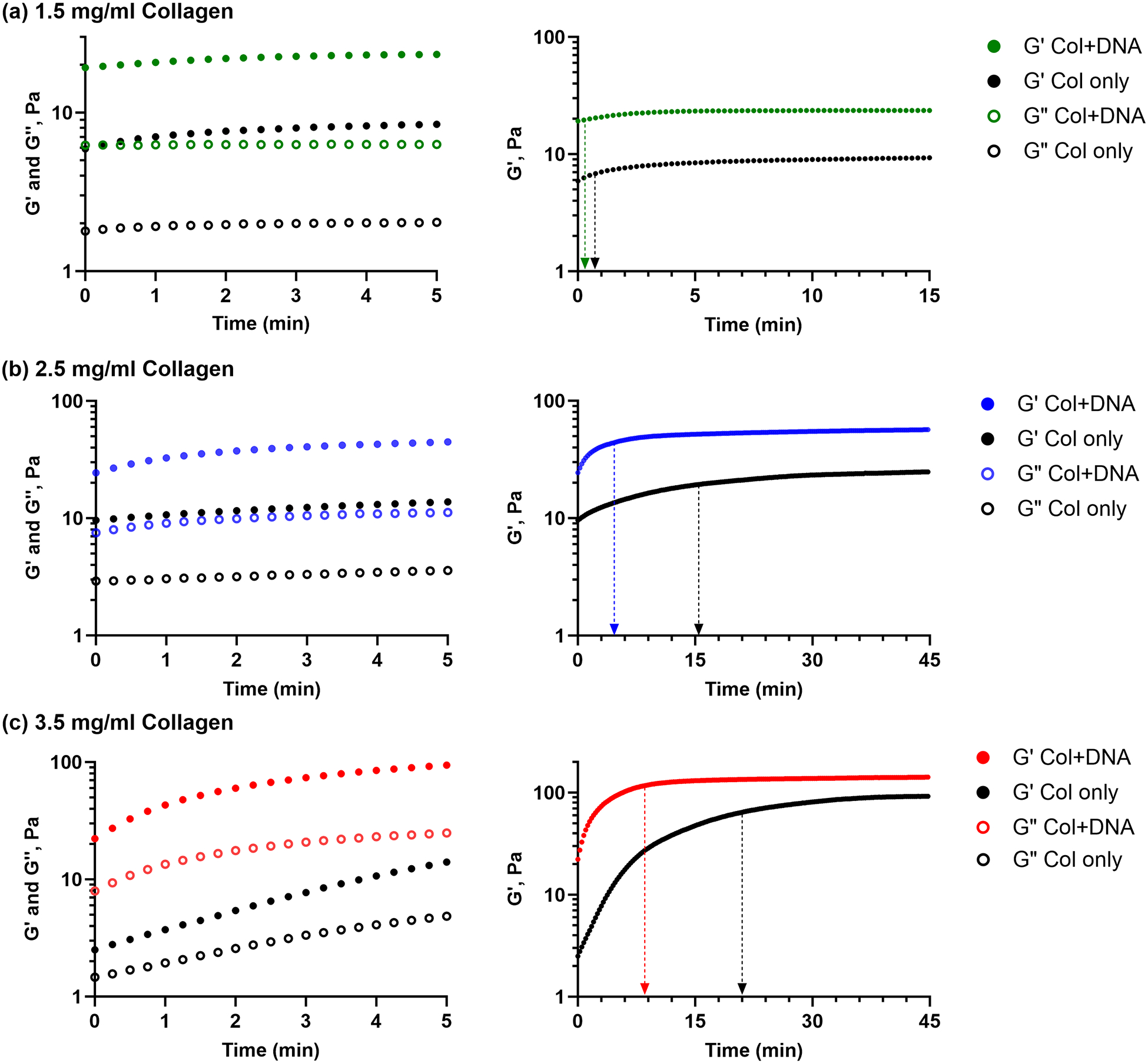
Gelation profiles of NACCs containing **a** 1.5 mg/mL collagen, **b** 2.5 mg/mL collagen, and **c** 3.5 mg/mL collagen through rheological monitoring of moduli at 25 °C. All values presented are averages of three independent measurements. The graphs on the left show both *G*′ (solid circles) and *G*″ (empty circles) and represent the first 5 min of gelation. Graphs on the right show only *G*′ for the entire experimental period of 45 min. Across all concentrations, the black line indicates *G*′ for collagen only (Col only), whereas colored lines indicate *G*′ for collagen in complex with DNA (Col + DNA). From the beginning, the consistent predominance of *G*′ over *G*″ suggests gel-like behavior, indicating structural integrity and varying degrees of stiffness and elasticity across samples. Arrows in the graphs to the right indicate the time point where gelation has occurred, as calculated with the sigmoidal curve based on the Hill equation

NACCs exhibit significant changes in rheological parameters within a short period, in contrast with other hydrogels, which may show more gradual changes over an extended time frame [21]. Our data reveals distinct gelation profiles for each sample, indicating commonalities and variations in their gelation kinetics (Fig. 2). For all examined collagen samples, regardless of the presence of DNA, the shear moduli exhibit an immediate increase without any detectable lag phase, indicating that a portion of gelation happened before the experiment could even be started. Right from the beginning, the elastic modulus surpassed the viscous modulus, indicating the presence of a stable, gel-like elastic network with no crossover between *G*′ and *G*″. In this case, samples containing collagen only (no DNA) display a longer gelation time, indicating a relatively slower gelation process. In contrast, samples with collagen and DNA exhibit shorter gelation times, indicating that the incorporation of ssDNA results in a faster gelation process compared to collagen alone. Gelation time was defined as the time required to reach half of the maximum *G*′, which was within 45 min for all gels.

All samples containing DNA exhibit faster gelation and higher modulus compared to the collagen-only control gels. Using the regression model described above, Fig. 2a shows that the *t*_*gel*_ has occurred for both samples containing 1.5 mg/mL collagen within 1 min (i.e., has started during sample loading and before the rheometer could start collecting data). Still, collagen with DNA reached that time faster than collagen only. Figure 2b shows the *t*_*gel*_ for 2.5 mg/mL collagen with DNA was < 5 min, compared to < 15 min for collagen only. In Fig. 2c, the *t*_*gel*_ for 3.5 mg/mL collagen and DNA was < 9 min, compared to < 20 min for collagen only. DNA accelerates gelation since the growth phase in hydrogels containing DNA is steeper and ends sooner, with the plateau region appearing earlier than the hydrogels without DNA.

### ssDNA Enhances the Material Properties of NACC Hydrogels

**Fig. 3:**
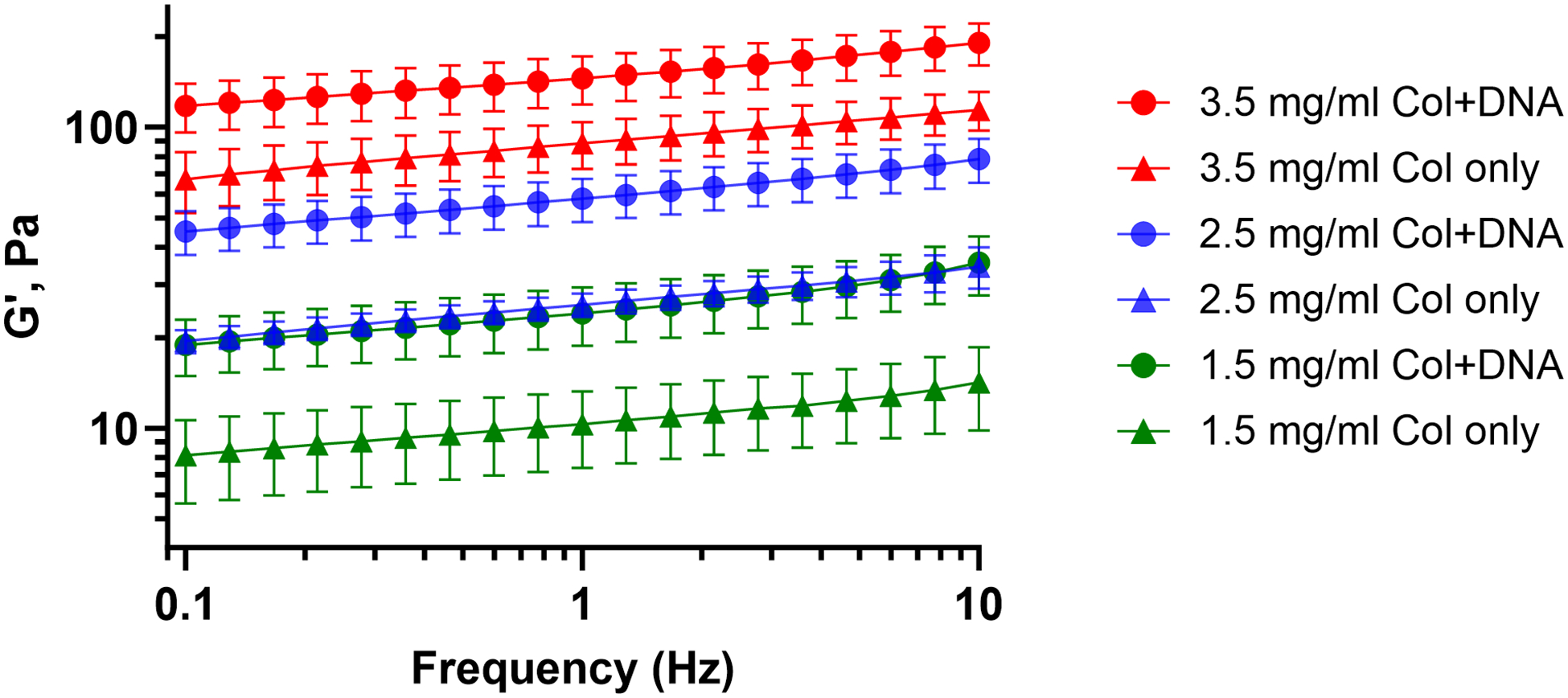
Evolution of storage modulus, *G*′, of hydrogels at different concentrations of collagen, with or without DNA. *G*″ is not shown. Increasing the concentration of collagen results in increased modulus, further amplified in the presence of DNA. Data are mean ± SD

**Fig. 4:**
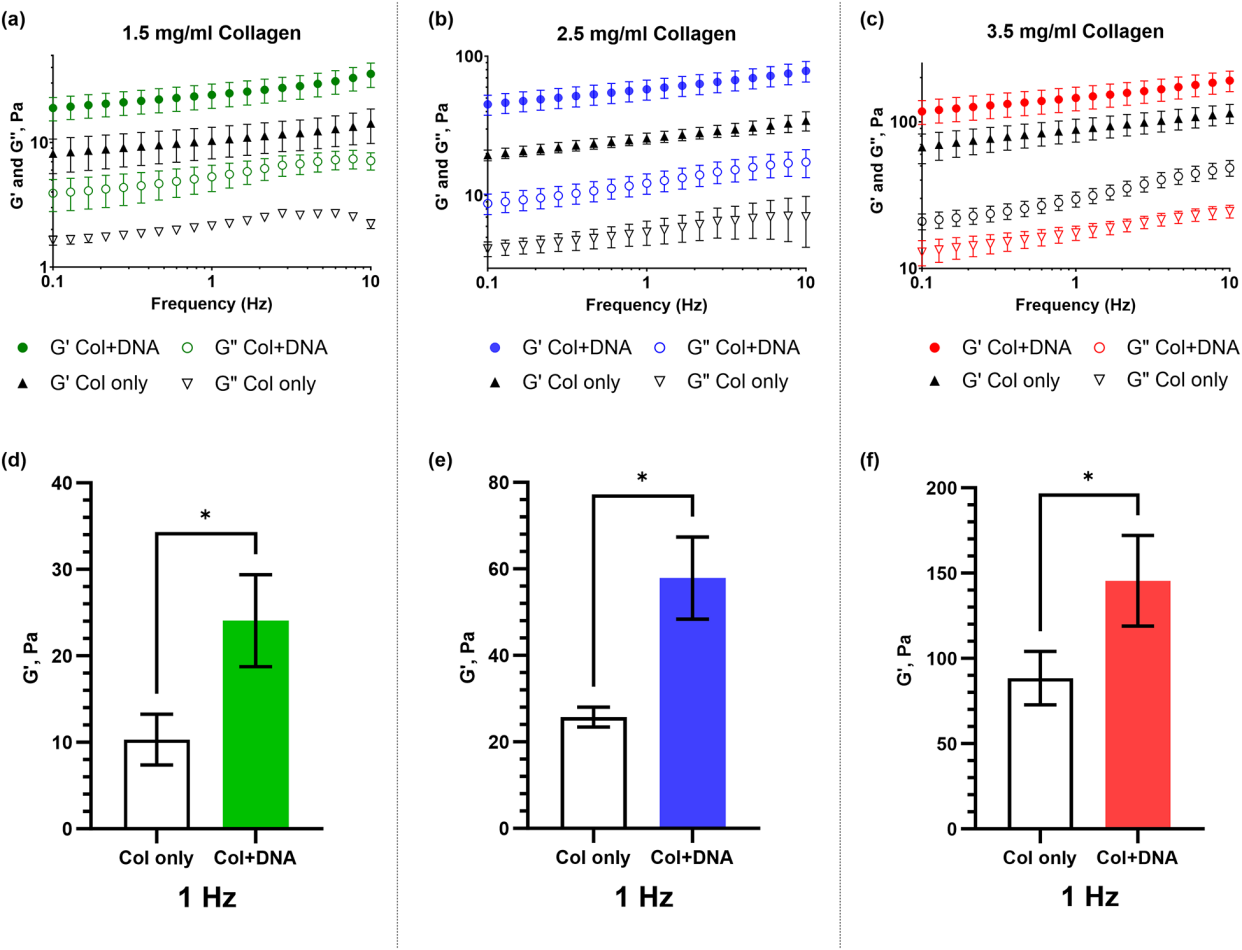
**a**–**c** Frequency sweeps of individual samples at the range of 0.1–10 Hz. **d**–**f** Bar graphs of *G*′ at a selected representative frequency of 1 Hz. Distinct differences in the measured stiffnesses can be seen across a range of frequencies tested. Error bars show mean ± SD. The *T*-test revealed a statistically significant difference among the means (*p* < 0.05), as indicated by the *p*-value summary (*)

The addition of DNA results in gels with higher moduli compared to the ones with collagen only (Fig. 3). A range of achievable elastic moduli (from less than 10 to more than 150 Pa) is demonstrated. The differences in the magnitudes of *G*′ among the samples are detailed in Fig. 4. DNA-containing samples exhibit the highest *G*′ values, indicating a more elastic behavior. On the other hand, samples with collagen only display lower *G*′. As can be seen, the moduli of gels containing DNA increase significantly compared to collagen alone. ssDNA oligonucleotides increase shear elastic modulus in the bulk gel while their relative influence is reduced with increasing collagen concentration. At 1.5 mg/mL (Fig. 4d), *G*′ increases from 10 to 24 Pa (+ 140%). At 2.5 mg/mL (Fig. 4e), *G*′ increases from 26 to 58 Pa (+ 123%). At 3.5 mg/mL (Fig. 4f), *G*′ increases from 88 to 146 Pa (+ 66%).

Nucleic acid aptamer length is usually in the range of 10 to 100 nucleotides [22]. To encompass a range of lengths, we also conducted a frequency sweep for samples containing shorter nucleotide sequences (Supplementary Fig. 3). Notably, the evolution of *G*′ exhibits a specific order, with the highest modulus observed for the 80-nucleotide sample, followed by the 20-nucleotide sample, with collagen only exhibiting the lowest modulus. This trend suggests that the nucleotide length also influences the stiffness and elastic behavior of the NACCs.

### ssDNA Affects Collagen Network Architecture and Serves as an Agglomerating Agent

**Fig. 5:**
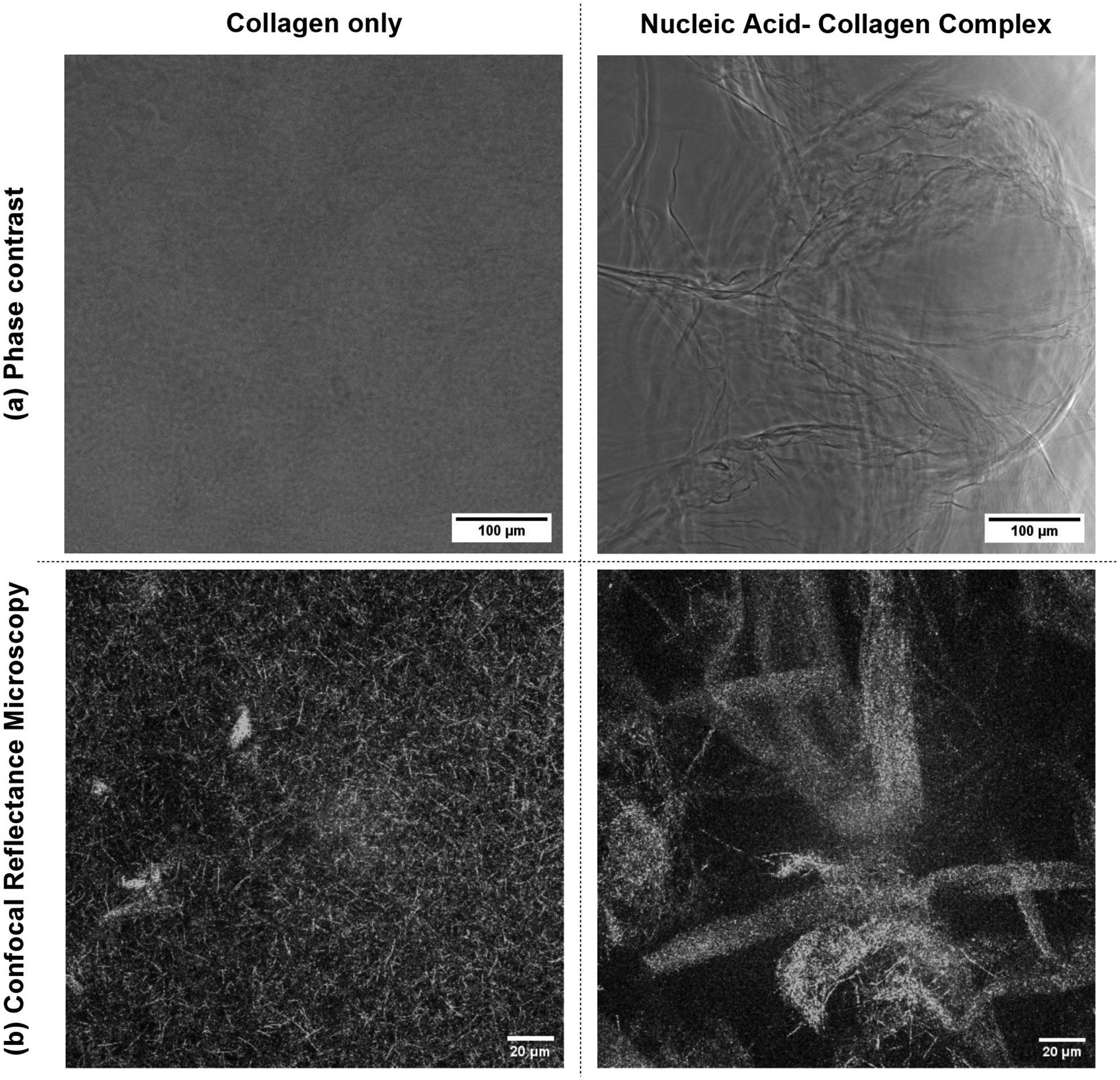
**a** Phase contrast demonstrated the formation of large, interconnected NACC fibers. In contrast to collagen only where individual fibrils are indistinguishable, the DNA-collagen fibers were also visible with the naked eye, offering resistance to a pipette tip upon touch. **b** Representative CRM images after complete gelation, at 2.5 mg/mL. Without DNA, the collagen network architecture, consisting of a dense network of cross-linked fibrils, seems typical. Upon the addition of DNA, a dramatic change occurs, with the formation of a system-spanning network of larger fibers, fiber bundles, and branches. Crosslinked fibers are morphologically transformed into an aggregate—adding DNA results in fibers with an average 10-fold increase in diameter

Phase contrast light microscopy images revealed the presence of randomly oriented fibers of various lengths in NACCs, in contrast to collagen alone, where the individual fibrils could not be distinguished (Fig. 5a).

Confocal reflectance microscopy visualization of collagen alone allowed us to observe a consistent and homogeneous distribution of microfibrils with an average fibril width of 1.342 ± 0.566 μm, a typical characteristic of collagen [23, 24] (Fig. 5b). In contrast, the observation upon the addition of DNA reveals a dramatically different network architecture, including a system-spanning arrangement of larger fibers and branches, with an average fiber bundle width of 12.773 ± 2.45 μm, which can be directly correlated with the observed increase in stiffness (Fig. 5b).

**Fig. 6:**
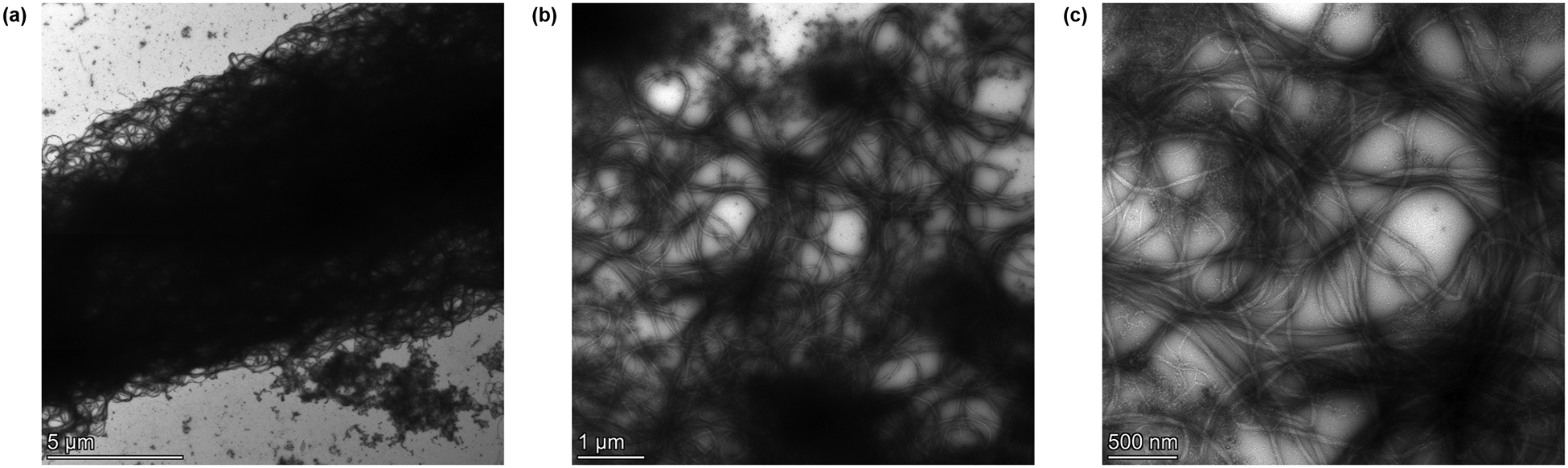
TEM verifies the presence of thick, bundled fiber segments in NACCs (negative staining conditions: aq. uranyl acetate 2% w/v, collagen concentration 0.25 mg/mL). **a** Despite being imaged 10 times more dilute than CRM, the width of a thick bundle remains at the same order of magnitude. The bundles themselves appear to consist of a dense network of individual collagen fibrils in an aggregated state induced by adding ssDNA (magnification: × 3400). Details of the aggregated fibrils at **b** × 8500 and **c** × 17,500 are also shown

Transmission electron microscopy was used to observe the thick and dense NACC fiber bundles at high magnification (Fig. 6). The TEM images reveal that the thick fiber segments comprise a mesh collection of individual fibrils. When ssDNA is introduced, they arrange in this pattern, with the average width of a bundle at 11.241 ± 1.02 μm, as measured at three different locations (Supplementary Fig. 1).

These results strongly suggest that DNA-collagen aggregates play a pivotal role in governing the mechanical properties of the hydrogel. We suspect that DNA serves as an agglomerating agent, controlling and promoting the complexation of fibrils into large bundles, which in turn increases the elastic modulus of the bulk hydrogel. ssDNA directs the assembly of collagen fibers, acting almost as a “molecular glue.”

## Discussion

Collagen has garnered significant attention in rheological investigations as a well-investigated natural polymer. Its viscoelastic properties have been extensively characterized, establishing collagen as a benchmark material for understanding the mechanical behavior of biological tissues [25]. Collagen fibril formation is a complex phenomenon that can vary between in vivo and in vitro contexts [26]. In vitro collagen fibril formation represents a distinct process from its in vivo counterpart, characterized by its relative simplicity [27]. The journey to fibril formation in vitro is not fixed, contingent upon various fibrilization conditions, and facilitated by the arrangement of sidechains on the helix surface, determined by amino acid sequences. Evidence from multiple studies highlights the inherent affinity of collagen for DNA (such as the formation of immune complexes by DNA binding to collagen and collagen-like structures within glomerular basement membranes [19]). Considering the interplay between DNA and collagen [28], our findings may offer insights into the potential mechanism underlying the role of DNA as a key player in collagen fibril formation and hydrogel mechanics under controlled in vitro conditions.

The established rheological understanding of collagen provides a solid foundation for investigating its behavior in hydrogel systems and emphasizes its suitability as a tunable component for developing advanced biomaterials. Our presented research builds upon rheological knowledge of collagen to explore the tunability of ssDNA-collagen gels. The collagen concentration in our NACCs was varied to evaluate the importance of DNA oligonucleotides in forming mechanically dynamic hydrogels. This approach can be applied to stiffness-dependent formation and maintenance of tissues, where microenvironmental composition can be adjusted to achieve defined matrix elasticity with the presence of distinct aptamer sequences. One notable application is the creation of ultra-soft microenvironments within the body, facilitating the study of delicate cellular processes. Among others, these hydrogels can offer ideal conditions for embryonic stem cells (ESCs) [29–31], early brain neural development and neurite branching [32–34], as well as cancer modeling [35–37]. To address the potential influence of GC content (i.e., the percentage of bases in the DNA sequence that are either guanine, G, or cytosine, C) on the bulk stiffness of NACCs, we performed frequency sweeps with sequences of variable GC contents (Supplementary Fig. 4) observing no significant differences among samples. This is in line with previous research suggesting that physicochemical features of nucleic acids are largely independent of the specific nucleotide sequence [38, 39] and underscores the versatility of DNA-collagen complexes as tunable biomaterials, offering consistent mechanical properties regardless of GC-content variations.

Collagens derived from multiple species can be used to culture various cell types. Still, they can substantially diverge in terms of 3D lattice fibril architecture and in-network stiffness, despite being used at equivalent concentrations [40, 41]. This allows for a great variety of matrix scaffold systems that can offer different in vitro morphological and biochemical properties [42], such as cell migration kinetics and expression of glycoproteins [43–45]. Thus, in addition to bovine type 1 collagen, we also identified the effect of ssDNA on collagen type 1 derived from rat-tail (Supplementary Figs. 5 and 6), observing similar findings. For rat-tail collagen alone, both *G*′ and *G*″ were very low (< 1 Pa), with *G*″ being above *G*′, suggesting a non-gelled, liquid-phase dominance. However, the addition of ssDNA clearly transforms the mix to a gel phase, a characteristic behavior of physical crosslinking suggesting the formation of a 3D network [46, 47]. Findings suggest that ssDNA oligonucleotides induce the formation of fibrous sheets, revealing that the addition of DNA to collagen leads to the emergence of a network that was previously non-existent in the absence of DNA. This observation aligns with our rheological finding, highlighting the transition from a liquid-phase dominance to a gel-like behavior.

The observed changes in *G*′ and *G*″ provide insights into the formation and strengthening of the gel structure, allowing for the determination of gelation kinetics and the establishment of a gel point. A gradual increase suggests ongoing structural changes (or network strengthening), while a plateau indicates that a material has reached a stable state. Despite the lack of a lag phase, the time sweep indicates the presence of a small growth phase followed by a plateau region. This agrees with previous findings on the behavior of type 1 collagen [48]. To comprehensively understand the NACCs’ dynamic behavior and viscoelastic nature, we examine frequency-dependent behavior and observe similar trends in the frequency sweeps across the different samples. Expectedly, as also demonstrated in previous studies [49], an increase in the concentration of collagen results in an increase in elastic and viscous moduli. We are the first to show that the presence of DNA further enhances the overall strength of the gel, suggesting that the internal microstructural rearrangement leads to robust reinforcement in the macroscale. Alterations in the structural organization of collagen (i.e., filament size) due to the addition of ssDNA significantly increase the shear elastic modulus. The emergence of new structures due to nucleic acid-collagen complexation affects the viscoelasticity of the system, by enhancing the overall mechanical integrity. Our FTIR-ATR data indicates that the interaction between collagen and ssDNA is purely supramolecular, with no significant hydrogen bonding as a primary driving force. Moreover, alteration of the chemical composition of either species through covalent modification can be excluded owing to the retention of all characteristic IR bands following complexation. We hypothesize that upon the addition of ssDNA, the supramolecular complexation and subsequent NACC formation are driven by hydrophobic interactions between the two biomacromolecules [50]. The presence of ssDNA decreases the number of individual “free-floating” collagen fibrils and triggers the emergence of large fibrous bundles, which appear to be an aggregated collection of individual collagen fibrils. This, in turn, increases the *G*′. Previous studies have also suggested changes in mechanical properties due to structural rearrangement of collagen [51, 52]; however, none of those investigations were focused on DNA-induced collagen structural modifications.

FTIR-ATR, rheology, and microscopy were used in a complementary manner to offer a detailed assessment of the structural changes that collagen undergoes upon the complexation induced by DNA. This attempts to bridge the gap between bulk mechanical behavior and microscale spatial arrangements, allowing for direct correlation with observed changes in stiffness. It is worth mentioning that for TEM, we had to use a very dilute collagen solution in order to be able to identify structures clearly; however, even with a 10-fold reduction in concentration, the width of the thick fiber segment was similar to the ones observed with CRM (Supplementary Fig. 1).

## Conclusions

The presented data comparing different DNA-collagen compositions provides insights into the behavior of these hydrogels and underscores the multifunctional role of DNA in biomaterial design. A versatile and tunable type of nucleic-acid collagen complex is demonstrated. The fibril structure of nucleic-acid collagen complexes was investigated using microscopy techniques, which indicate the ssDNA-directed assembly of collagen fibers. The addition of ssDNA oligonucleotides promotes the rearrangement of the crosslinked pattern of the collagen fibrils into larger and aggregated fibers, which can be attributed to the observed increase in stiffness as measured by rheology. The observed amplification of amide bands in FTIR-ATR spectra and the emergence of a distinct phosphate backbone peak strongly support a purely supramolecular interaction between collagen and ssDNA, affirming the unaltered chemical composition of both species during complexation.

Overall, our results highlight the viscoelastic behavior of NACCs and the ability to modulate their mechanical properties based on the presence of oligonucleotides. This is a novel approach to leverage DNA as a tunable characteristic in collagen-based hydrogels. The results clearly suggest that fibrillogenesis and the internal microstructure of NACCs are dependent on the DNA oligonucleotide length and molar ratio. Structure and mechanical properties can be effectively controlled using various types of collagens. The exploration of DNA-induced tunability in collagen-based hydrogels represents an advancement in the design of bioactive materials with tailored properties. By harnessing the unique properties of DNA, we can develop a customizable material system that closely mimics anatomically relevant environments in terms of stiffness, topography, and biochemistry.

This technology has the potential to facilitate reproducible studies of native cellular functions and promote advancements in biomedical research. While previous work has demonstrated the utilization of DNA crosslinking in other gel systems, our study explores the unique mechanical behaviors induced by DNA-collagen interactions, offering insights into the specific context of DNA-collagen hydrogel systems and their potential in diverse biomedical applications. Nucleic acid-collagen hydrogels open new avenues for understanding complex biological phenomena and developing innovative approaches for regenerative medicine, drug delivery, and tissue engineering by providing a biomimetic platform.

## Supplementary Material

Supplemental File

## Figures and Tables

**Table 1:** DNA sequences

DNA oligonucleotide length (nt) and molecular weight	Sequence
80 nt Mw = 24,681 g/mol	5′–AATATCTCGCGCGATAGCGATCGACTAGCTGAGCTATGCTAGCAACTGACATACTGAGCTAGCCTGAACGTGACTGAACG–3′
20 nt Mw = 6117 g/mol	5′–AATCTGCGATCGATCGATGC–3′

**Table 2:** Molar ratios of collagen (Col) to DNA. Control samples were collagen at the above concentrations (1.5 mg/mL, 2.5 mg/mL, 3.5 mg/mL) without the presence of ssDNA

Reagent	Molarity (μM)	Molar ratio (Col: DNA)
1.5 mg/mL collagen type 1	5	0.5:1
ssDNA	10	
2.5 mg/mL collagen type 1	8.33	0.833:1
ssDNA	10	
3.5 mg/mL collagen type 1	11.67	1.167:1
ssDNA	10	

## Data Availability

The authors confirm that the data supporting the findings of this study are available within the article and its supplementary materials.
