# Supplementary material for: Toward Bioactive Hydrogels: A Tunable Approach via Nucleic Acid-Collagen Complexation
Source: Regen Eng Transl Med. Author manuscript; Available in PMC 2025 Jul 27. (PMC12291172; doi:10.1007/s40883-024-00345-1)
Supplement: Supplemental File [file NIHMS2025316-supplement-Supplemental_File.docx]

# **Towards Bioactive Hydrogels: A Tunable Approach via Nucleic Acid-Collagen Complexation**

Nikolaos Pipis^1^, Senthilkumar Duraivel^2^, Vignesh Subramaniam^3^, Kevin A. Stewart^4^, Thomas E. Angelini^3^, Josephine B. Allen^1,2^*

^1^ J. Crayton Pruitt Family Department of Biomedical Engineering, University of Florida, Gainesville, Florida 32611, USA

^2^ Department of Materials Science & Engineering, University of Florida, Gainesville, Florida 32611, USA

^3^ Department of Mechanical and Aerospace Engineering, University of Florida, Gainesville, Florida 32611, USA

^4^ George & Josephine Butler Polymer Research Laboratory, Department of Chemistry, Center for Macromolecular Science & Engineering, University of Florida, Gainesville, FL 32611, USA

* Corresponding Author

Josephine Allen, Ph.D.

Professor

jallen@mse.ufl.edu

**Supplementary Figures**

__________________________________________________________________________________________________


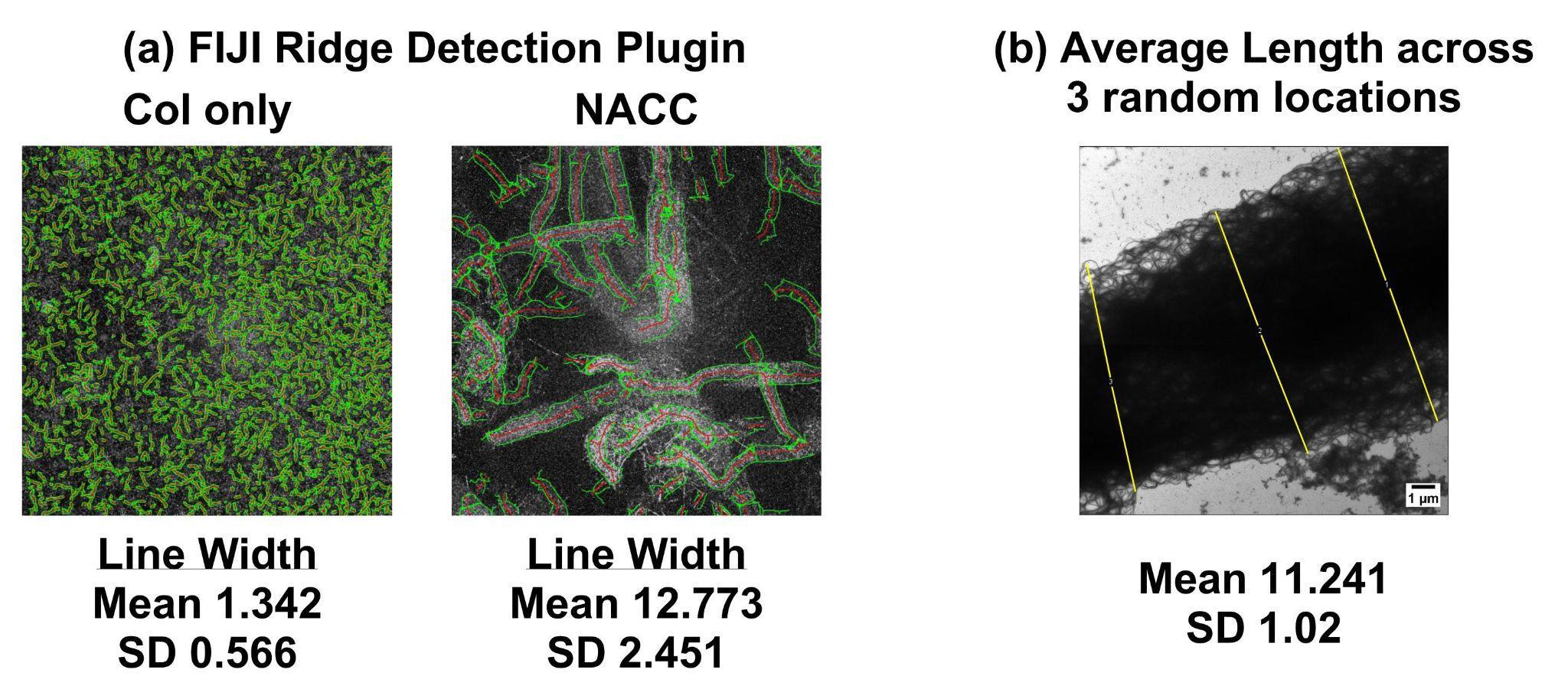


**Supp. Fig. 1 (a)** FIJI Ridge Detection Algorithm for calculating fibril and fiber width from the CRM images **(b)** Width measurement across a thick fiber from TEM image.


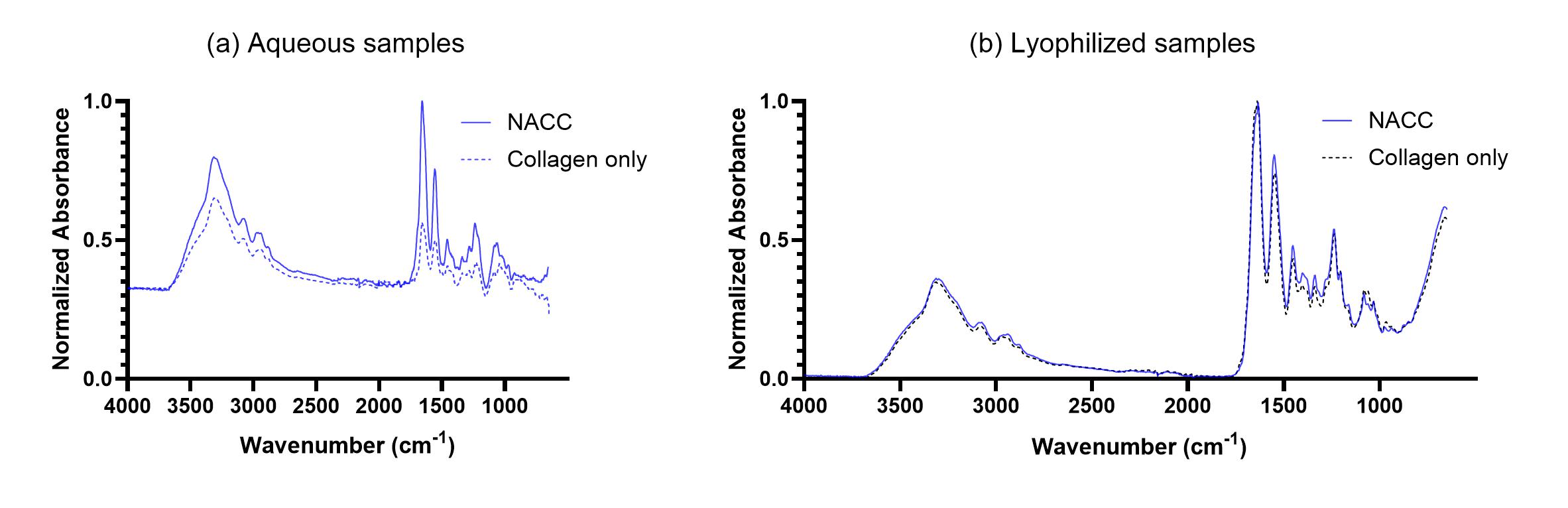


**Supp. Fig. 2** FTIR-ATR along the full spectrum, 4000-650 cm^-1^, to illustrate the unchanged position of Amides A and B in the 3000-3500 cm^-1^ region, as well Amides I, II, III in the 2000-650 cm^-1^ region **(a)** Aqueous sample (from main text Figure 1) **(b)** Dry state samples following lyophilization. The unchanged peak positions in the 3200-3500 cm^-1^ region suggests no significant intermolecular hydrogen-bonding between the ssDNA and collagen (no peak shifts).


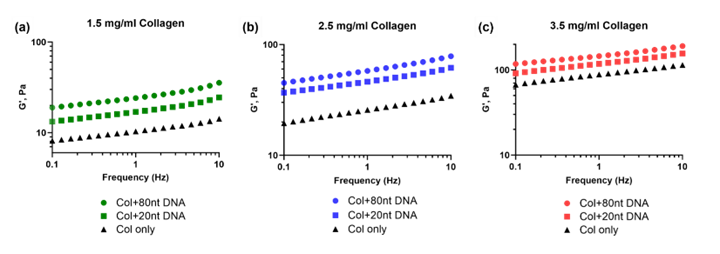


**Supp. Fig. 3** Frequency sweep analysis of varying DNA lengths, highlighting the impact of nucleotide length on the viscoelastic properties of NACCs. For all samples (**(a)** 1.5 mg/ml, **(b)** 2.5 mg/ml and **(c)** 3.5 mg/ml), the modulus of the gel containing DNA with 80 nucleotides surpasses the modulus of the gel containing DNA with 20 nucleotides, which in turn surpasses the modulus of the collagen alone.

| GC-content | Sequence | Mw (g/mol) | *G’* at 1 Hz (Pa), mean ± SD |
| --- | --- | --- | --- |
| 0% (Control, shown in main text) | N/A | N/A | 10.315 ± 2.932 |
| 10% | 5’ – *TAAAATTTATATAAAATTTTATTAATAAATTTTTATAAACTTAATATATTTCTTAATATAACACGACATATGTAAATTAG* – 3’ | 24,607 | 23.377 ± 2.126 |
| 25% | 5’ – *AATCTATGCAATATATATATCGACCCAATTTTATATCTCAGTTAAGCTGAGCAGAATGATCTCAATATATTTAAAAATTA* – 3’ | 24,582 | 20.770 ± 3.562 |
| 50% (shown in main text) | 5’ – AATATCTCGCGCGATAGCGATCGACTAGCTGAGCTATGCTAGCAACTGACATACTGAGCTAGCCTGAACGTGACTGAACG – 3’ | 24,681 | 24.082 ± 5.316 |
| 75% | 5’ – *TCCCTATGCGCGCGCGCGCGACCCGGGGACGCGCGCGGCGGCCCGCGGCGCGCGAATGATCTCGAGATCCACACACGGTA* – 3’ | 24,641 | 27.627 ± 3.123 |
| 90% | 5’ – *TCCCTATGCGCGCGCGCGCGACCCGGGGACGCGCGCGGCGGCCCGCGGCGCGCGCGCGCGCGCGCGAGCCGCGCACGGCC* – 3’ | 24,638 | 26.655 ± 1.170 |

**Supp. Fig. 4** Our results reveal that among samples with varying GC-content, all exhibited stiffness values within the same range. Shown are the samples of 1.5 mg/ml collagen concentration. This finding is in line with existing research and suggests that the physicochemical properties of these complexes, with respect to their bulk stiffness, remain largely independent of their specific nucleobase sequences.


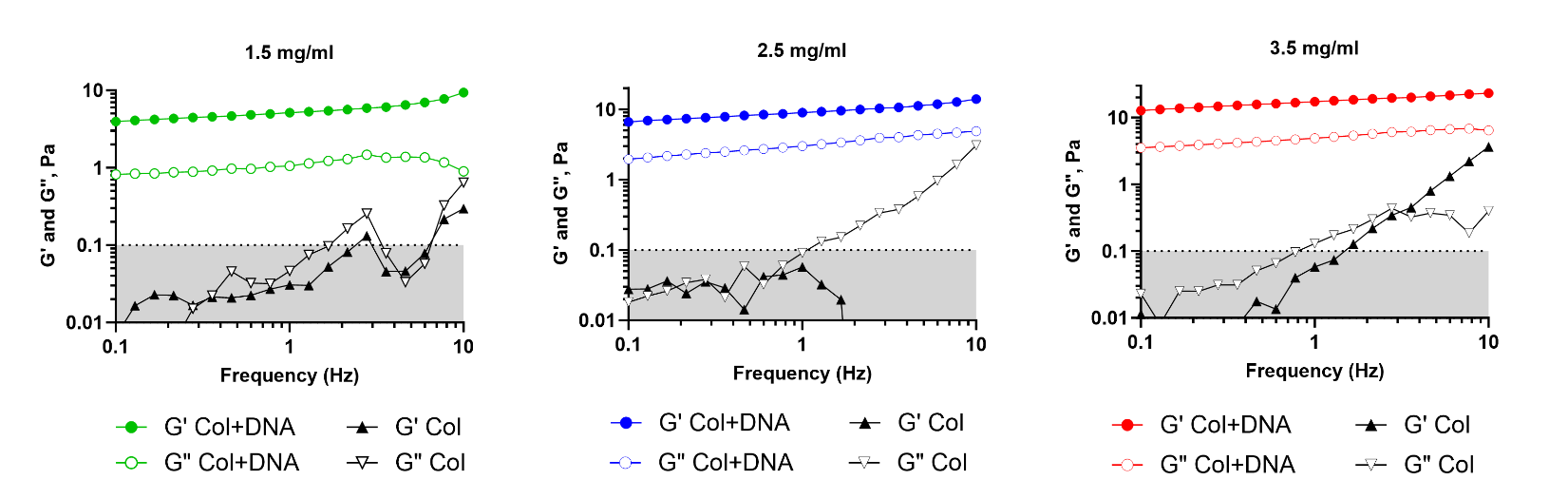
**Supp. Fig. 5** Rheological findings from rat-tail collagen. The dominance of *G’* over *G”* in the DNA-containing samples indicates the development of a collagen fibril structure. In collagen alone, the relationship of *G’* with *G”* is unclear. Values in the grey area of the graph were considered noise.

**
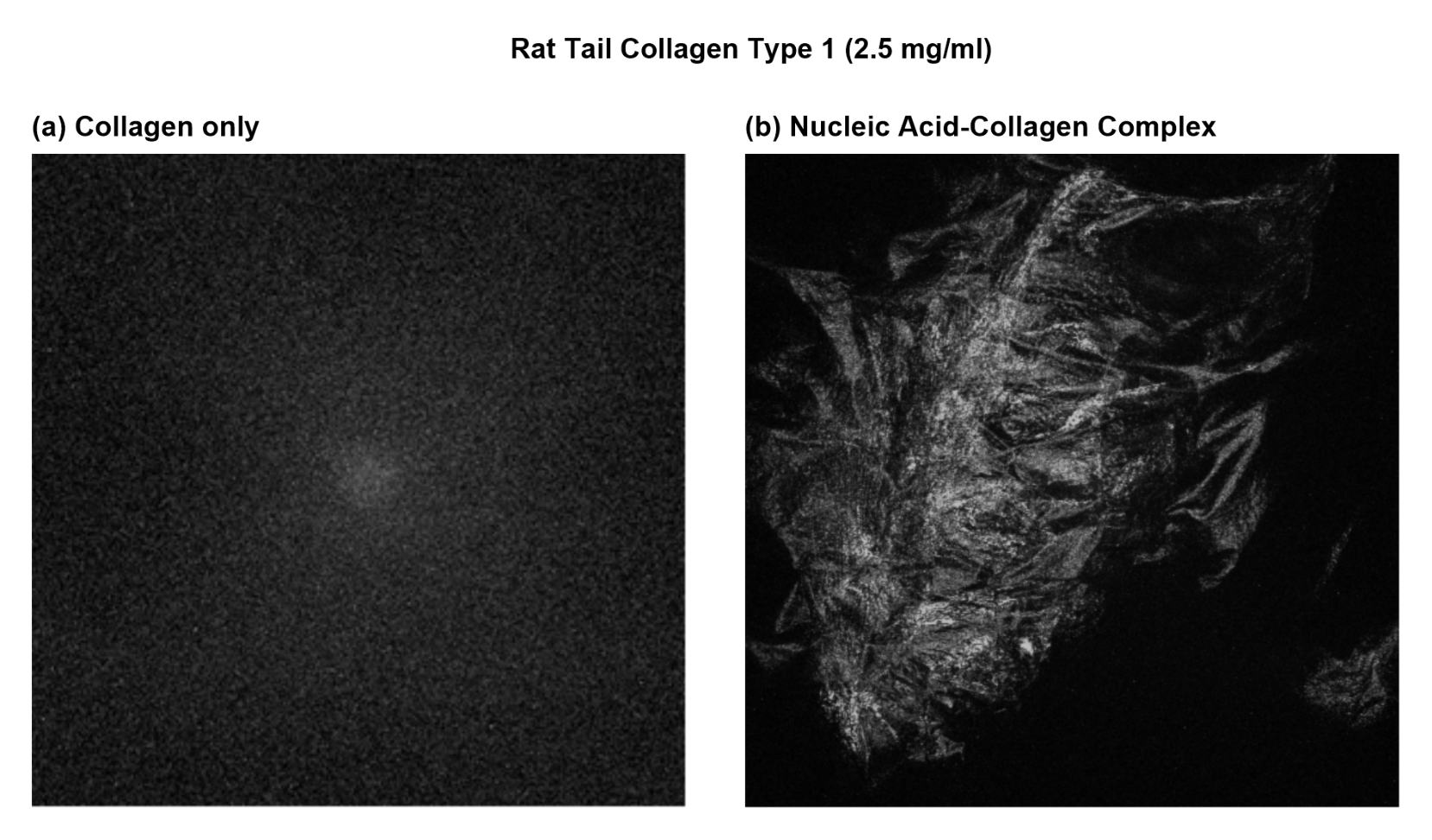
**

**Supp. Fig. 6** Representative confocal reflectance images of rat-tail type 1 collagen, 2.5 mg/ml **(a)** No structures are visible due to the liquid-dominant nature at that concentration **(b)** A clear induction of sheet-like fibril formation is demonstrated upon addition of ssDNA. Findings show that DNA induces the formation of a fibrous sheet, revealing that the addition of DNA leads to the emergence of a network that was previously non-existent in the absence of DNA. This observation aligns with our rheological finding, highlighting the transition from a liquid-phase dominance to a gel-like behavior.
